# Enhancer polymorphisms at the *IKZF1* susceptibility locus for acute lymphoblastic leukemia impact B-cell proliferation and differentiation in both Down syndrome and non-Down syndrome genetic backgrounds

**DOI:** 10.1371/journal.pone.0244863

**Published:** 2021-01-07

**Authors:** Vincent U. Gant, Jacob J. Junco, Maci Terrell, Raushan Rashid, Karen R. Rabin

**Affiliations:** Department of Pediatrics, Section of Hematology-Oncology, Baylor College of Medicine, Houston, Texas, United States of America; Hirosaki University Graduate School of Medicine, JAPAN

## Abstract

Children with Down syndrome have an approximately 10-fold increased risk of developing acute lymphoblastic leukemia and this risk is influenced by inherited genetic variation. Genome-wide association studies have identified *IKZF1* as a strong acute lymphoblastic leukemia susceptibility locus in children both with and without Down syndrome, with association signals reported at rs4132601 in non-Down syndrome and rs58923657 in individuals with Down syndrome (r^2^ = 0.98 for these two loci). Expression quantitative trait locus analysis in non-Down syndrome lymphoblastoid cell lines has demonstrated an association between the rs4132601 risk allele and decreased IKZF1 mRNA levels. In this study, we provide further mechanistic evidence linking the region encompassing *IKZF1*-associated polymorphisms to pro-leukemogenic effects in both human lymphoblastoid cell lines and murine hematopoietic stem cells. CRISPR/Cas9-mediated deletion of the region encompassing the rs17133807 major allele (r^2^ with rs58923657 = 0.97) resulted in significant reduction of IKZF1 mRNA levels in lymphoblastoid cell lines, with a greater effect in Down syndrome versus non-Down syndrome cells. Since rs17133807 is highly conserved in mammals, we also evaluated the orthologous murine locus at rs263378223, in hematopoietic stem cells from the Dp16(1)Yey mouse model of Down syndrome as well as non-Down syndrome control mice. Homozygous deletion of the region encompassing rs263378223 resulted in significantly reduced Ikzf1 mRNA, confirming that this polymorphism maps to a strong murine *Ikzf1* enhancer, and resulted in increased B-lymphoid colony growth and decreased B-lineage differentiation. Our results suggest that both the region encompassing rs17133807 and its conserved orthologous mouse locus have functional effects that may mediate increased leukemia susceptibility in both the Down syndrome and non-Down syndrome genetic backgrounds.

## Introduction

Genome-wide association studies (GWAS) of childhood acute lymphoblastic leukemia (ALL) have identified risk loci at several genes involved in B cell differentiation, including *IKZF1*, *CDKN2A*, *ARID5B*, *CEBPE*, *GATA3*, *BMI1*, and *PIP4K2A* [1–6]. We recently conducted the first GWAS of ALL in children with Down syndrome (DS), who have an approximately 10-fold increased risk of developing ALL compared to children without DS [7, 8], and determined that several of the same loci also influence ALL risk in DS [9]. We identified the single-nucleotide polymorphism (SNP) rs58923657 near *IKZF1* as one of the top association signals (odds ratio, 2.02; *P* = 5.32 x 10^−15^) and determined that the rs58923657 haplotype block maps to a super-enhancer in B cells. We further demonstrated that the risk allele for rs58923657 and nearby SNPs in linkage disequilibrium (LD; r^2^ > 0.6) are associated with significantly decreased enhancer activity and differential nuclear protein binding [9]. However, the mechanism underlying the association between *IKZF1-*associated SNPs and ALL susceptibility, and whether it differs in the DS genetic background, remains unclear.

*IKZF1* encodes Ikaros, a zinc finger transcription factor and master regulator of lymphoid differentiation [10]. *IKZF1* positions chromatin remodeling complexes near lymphoid genes to modulate their expression in hematopoietic stem cells (HSCs) [11]. Somatic alterations in *IKZF1* are frequent in B-ALL, typically affect the DNA-binding domain resulting in loss of function, and are associated with poorer prognosis [12, 13]. Genetic loss of Ikaros in pre-B lymphocytes results in arrested B cell differentiation and increased proliferation [14]. The relative expression levels of *IKZF1* influence normal B cell development and resistance to transformation. Mice carrying a hypomorphic *Ikzf1* allele show significantly reduced Ikaros levels and exhibit impaired B cell production and reduced differentiation in pre-B lymphocytes [15]. Although reduced *Ikzf1* expression alone did not lead to spontaneous B-ALL, these *Ikzf1* haplodeficient mice exhibited accelerated onset of B-ALL when combined with the *BCR-ABL1* transgene [16].

The majority of disease-associated SNPs identified in GWAS map to noncoding regions of the genome and are predicted to influence distal gene expression [17–20]. Additionally, these SNPs are more frequently located in super-enhancers of the disease-associated tissues [21]. Super-enhancer domains consist of clusters of typical enhancers and are found near master regulator genes [22]. These studies suggest that noncoding SNP variation contributes to disease risk by influencing the activity of overlapping regulatory regions and modulating master regulator gene expression in an allele-specific manner. Indeed, allele-specific regulatory changes associated with noncoding leukemia risk loci have been observed [6, 23–25]. Expression quantitative trait locus analysis of lymphoblastoid cell lines (LCLs) shows germline variation in *IKZF1* is associated with significant allele-specific decreases in *IKZF1* expression [1]. However, the identity of the regulatory regions influenced by *IKZF1* risk SNPs and the manner in which they mediate ALL risk are unclear.

In this study, we functionally characterized the *IKZF1* risk locus and compared the effects in the DS and non-DS genetic backgrounds, to identify effects that may mediate B-ALL susceptibility. We used CRISPR/Cas9 in patient-derived LCLs to generate isogenic clones carrying biallelic deletions of candidate functional *IKZF1* risk SNPs, and then examined *IKZF1* expression in the SNP deletion clones to assess the enhancer potential of each region. We demonstrate that the region encompassing rs17133807 exhibits *IKZF1* transcriptional enhancer activity and is conserved in mice. We used CRISPR/Cas9 to delete the murine orthologue to the rs17133807 enhancer region in HSCs from the Dp16(1)Yey [26] (henceforth referred to as Dp16) mouse model of DS, and in non-DS littermate control mice, in order to further assess the functional impact of this locus on B cell proliferation and differentiation.

## Materials and methods

### Cell lines and cell culture

Epstein-Barr virus-transformed LCLs were generated using blood samples from patients treated at Texas Children’s Hospital and genotyped for SNPs in the rs58923657 LD block as previously described [9]. Samples from patients were obtained with written informed consent according to protocols approved by the Institutional Review Board of Baylor College of Medicine. LCLs were maintained in RPMI 1640 medium supplemented with 15% FBS and 1% penicillin-streptomycin at 1.5–5 x 10^5^ cells/mL at 37°C and 5% CO_2_. HEK-293T [27] and NIH-3T3 cells [28] were maintained in DMEM with 10% FBS and 1% penicillin-streptomycin. HEK-293T and NIH-3T3 cells were kindly provided by Daniel Lacorazza (Baylor College of Medicine). OP9 stromal cells [29] were maintained between 40–60% confluency in Alpha MEM (ThermoFisher, Waltham, MA) supplemented with 20% FBS and 1% penicillin-streptomycin. OP9 stromal cells were kindly provided by Margaret Goodell (Baylor College of Medicine).

### Guide RNA design and plasmid construction

Dual guide RNAs (gRNA) with targets flanking the human SNPs rs62445866, rs6964969, rs6944602, rs10264390, and rs17133807, and flanking the enhancer encompassing murine SNP rs263378223 (mm9; chr11: 11,676,896–11,677,114) were designed using CRISPOR (http://crispor.tefor.net/) [30] and CHOPCHOP (https://chopchop.cbu.uib.no/) [31] design tools. Control non-targeting gRNA sequences expressed in mouse HSCs were selected from the Asiago mouse genome-wide gRNA library [32]. Off-target analysis for all gRNA sequences was conducted using the Cas-OFFinder webtool (http://www.rgenome.net/cas-offinder/) [33].

Genome editing was conducted using the pCLIP-All lentiviral vector system (TransOMIC technologies, Huntsville, AL), which expresses a gRNA, Cas9, and a fluorescence protein marker (ZsGreen or RFP). Complementary oligos encoding gRNA protospacer sequences (S1 Table) were hybridized in a thermal cycler by heating to 95°C for 5 min and cooling 5°C/min to 25°C. Hybridized gRNA oligos were cloned into BsmBI-digested pCLIP-All vectors. For simplicity, 5’-targeting gRNAs were cloned into pCLIP-All-ZsGreen and 3’-targeting gRNAs were cloned into pCLIP-All-RFP. Vector reconstructions were confirmed by Sanger sequencing using pCLIP-seq primer (S2 Table).

### Transfections and CRISPR/Cas9 editing in LCLs

LCLs were passaged to 2.5 x 10^5^ cells/mL 24 hours prior to transfection. Cells were washed twice in PBS (-Ca^2+^ -Mg^2+^) and 1.0 x 10^6^ cells were transfected with 4 ug of each pCLIP-All-gRNA vector. Cells transfected with empty pCLIP-All-ZsGreen and pCLIP-All-RFP were used as single-color controls for sorting. Transfections were performed with a Neon Transfection System (ThermoFisher) using 100 μL tips. Transfections were performed using buffer R with 2 x 1350V x 20 ms pulses. Transfected cells were dispensed into wells with pre-warmed media lacking pen/strep and incubated overnight. Cells were then washed in PBS and cultured in fresh complete media.

At 48 hours after transfection, cells were washed twice in PBS and resuspended in sorting buffer (PBS supplemented with 5% FBS, 1% penicillin/streptomycin). ZsGreen^+^/RFP^+^ cells were analyzed with FACSDiva software (Beckton Dickinson, Franklin Lakes, NJ) and sorted using a FACSAria Fusion cell sorter (BD Biosciences, San Jose, CA). Sorted cells were allowed to recover for at least 48 hours in medium supplemented with 20% FBS. Monoclonal cultures established from single-cells were prepared by diluting cells to 20 cells/mL and plating 200 μL/well into round-bottom 96-well plates. Plates were spun down at 300g x 5 min and single cells were confirmed by microscopy. Monoclonal cultures were expanded for at least 20 days.

### Mouse hematopoietic stem cells

Dp16 mice were obtained from The Jackson Laboratory. Dp16 and wild-type (WT) littermate control mice between 8–12 weeks old were injected intraperitoneally with 150 mg/kg of 5-fluorouracil (Sigma) in normal saline. After 5 days, mice were euthanized using CO_2_ followed by cervical dislocation and both legs were dissected in a biosafety cabinet. Bone marrow (BM) HSCs were flushed from both femurs and tibiae with HBSS+ (HBSS supplemented with 5% FBS, 1% penicillin-streptomycin, 250 ng/mL amphotericin B, filter-sterilized). Cells were dissociated by vortexing and passing through a 20G needle twice. Dissociated cells were strained with a 40-um filter. HSCs were maintained in X-VIVO 15 (Lonza, Basel, Switzerland) supplemented with 100 ng/mL SCF, 10 ng/mL IL-3 and IL-6 (PeproTech, Rocky Hill, NJ) until transduction. This study was carried out in strict accordance with the recommendations in the Guide for the Care and Use of Laboratory Animals of the National Institutes of Health. The protocol was approved by the Baylor College of Medicine Institutional Animal Care and Use Committee. All bone marrow harvests were performed on euthanized mice, and all efforts were made to minimize suffering.

### Lentiviral transductions and CRISPR/Cas9 editing in mouse HSCs

Lentivirus was generated by transfecting psPAX2 and pMD2.G plasmids (Addgene) and pCLIP-All-gRNA into HEK-293T cells with Lipofectamine 3000. Lentivirus supernatants were harvested after 48 hours of transfection and concentrated 200-fold by ultracentrifugation at 70,000g x 2 hours at 4°C. HSCs were transduced with lentiviruses by spinfection at 400g x 45 min at room temperature on Retronectin-coated plates (Takara, Mountain View, CA). Cells were collected and media was replaced with X-VIVO 15 supplemented with 100 ng/mL SCF, 10 ng/mL IL-3 and IL-6. For analysis of genome editing and gene expression in transduced HSCs, c-Kit^+^Sca-1^+^CD34^-^ZsGreen^+^RFP^+^ cells were sorted using a SH800S Cell sorter (Sony Biotechnology, San Jose, CA).

### Confirmation of CRISPR/Cas9 genome editing

Cell pellets were lysed in DNA extraction buffer (320 mM sucrose, 10 mM Tris-HCl, ph 7.5, 5 mM MgCl_2_, 1% Triton X-100) with 60 μg/mL proteinase K at 55°C. DNA was ethanol precipitated and quantitated. Samples were screened by PCR using primers flanking the targeted regions. All PCR reactions were performed using HotStarTaq DNA Polymerase (QIAGEN, Hilden, Germany). PCR products were cloned into the TA-cloning vector pGEM-T Easy (Promega) and sequenced to confirm genome editing. The sequences for primers used in this study are listed in S2 Table.

### Quantitative reverse-transcription polymerase chain reaction (qRT-PCR)

Total cellular RNA was extracted using Direct-zol RNA MicroPrep kits (Zymo Research, Irvine, CA). Synthesis of cDNA was performed using SuperScript IV VILO Master Mix (ThermoFisher) with both random hexamers and oligo-dT primers. qRT-PCR was performed using IKZF1 (Hs00958474_m1), B2M (Hs00187842_m1), Ikzf1 (Mm01187877_m1), and B2m (Mm00437762_m1) TaqMan Gene Expression assays (ThermoFisher) with TaqMan Fast Advanced Master Mix (ThermoFisher), according to the manufacturer’s instructions. Reactions were performed in technical triplicates using a StepOnePlus Real-Time PCR system (Applied Biosystems) measuring relative gene expression differences with the ΔΔCt method.

### Epigenetic annotation and conservation of *cis*-regulatory elements

Epigenetic profiling of the rs58923657 haplotype block was performed as previously described [9]. Epigenetic profiling of mouse *Ikzf1* and chromosome 11 was performed using ENCODE Project data [20] from DNase-sequencing (DNase-seq), and transcription factor and histone modification ChIP-sequencing (ChIP-seq) experiments. We examined mammalian sequence conservation at SNPs in the haplotype block using the UCSC genome browser [34].

### Lymphoid colony forming assays

For colony forming assays, transduced HSCs were washed with IMDM supplemented with 2% FBS (STEMCELL Technologies, Vancouver, Canada) and suspended in MethoCult 3630 semisolid medium (STEMCELL Technologies) supplemented with 20 ng/mL SCF and 250 ng/mL amphotericin B. The cell suspension was plated at 5 x 10^4^ cells per well in 24-well plates and incubated for 7 days. ZsGreen^+^/RFP^+^ colonies were counted using fluorescence microscopy.

### Stromal cell co-culture and hardy fraction analysis

For analysis of B cell differentiation, 5 x 10^4^ transduced HSCs were transferred to flasks of OP9 stromal cells and co-cultured in B lymphoid-promoting medium (Alpha MEM supplemented with 20% FBS, 1% penicillin-streptomycin, 50 μM 2-mercaptoethanol, 10 ng/mL SCF, 10 ng/mL Flt3L and 10 ng/mL mIL-7 (PeproTech, Rocky Hill, NJ), and 250 ng/mL amphotericin B). Cells were harvested by trypsinization and resuspended in sorting buffer after 7 days of co-culture. Hardy fraction analysis of B cell differentiation was performed as previously described [35]. Briefly, cells were stained with 7-AAD (eBioscience) and the following antibodies: B220-Pacific blue, CD43-APC, BP-1-BV605, CD24-PE-Cy7, IgD-APC-Cy7, and IgM-PE-CF594 (BD Biosciences). Single-color stained cells and UltraComp beads (eBioscience) were used for compensation and to establish Hardy fraction gating. The following controls were used to exclude OP9 cells and non-transduced cells from ZsGreen^+^/RFP^+^ analysis: OP9 cells alone, non-transduced HSPCs alone, and OP9 cells with non-transduced HSPCs. Samples were analyzed on an LSR II flow cytometer and FACS plots were generated using FlowJo software.

### Statistical analysis

Data for B220^+^ cell growth were analyzed using Student’s t-test. Gene expression, colony counts, and Hardy fraction data were analyzed using two-way ANOVA with Tukey’s multiple comparisons test. Statistical analyses were performed using GraphPad Prism (ver. 6.07).

## Results

### Functional analysis of regions harboring candidate causal SNPs in the *IKZF1* risk locus

**Fig 1:**
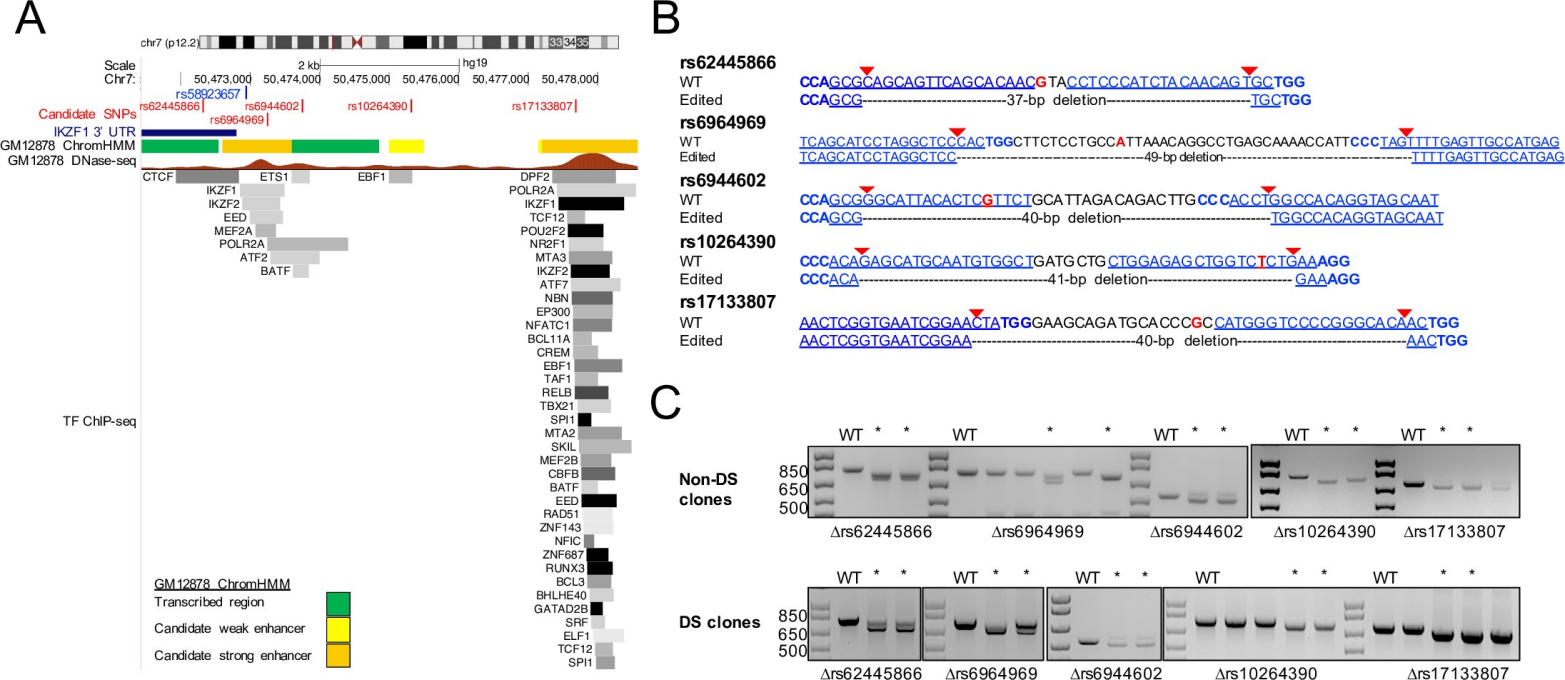
Targeting the rs58923657 haplotype block for CRISPR/Cas9-mediated SNP microdeletions in human LCLs. (A) Epigenetic profile of the region harboring the rs58923657 haplotype block. Tracks show the position of the *IKZF1* 3’ UTR (dark blue rectangle); the positions of rs58923657 (blue) and SNPs targeted for deletion (red); GM12878 chromatin state (ChromHMM); DNase-seq signal from GM12878 with signal strength corresponding to chromatin accessibility; and transcription factor ChIP-seq data from GM12878 and other ENCODE LCLs with darkness corresponding to signal strength. Data are provided by the ENCODE project and displayed in the UCSC genome browser (http://genome.ucsc.edu/). (B) Regions targeted for CRISPR/Cas9 deletion. The wild-type (WT) sequences correspond to the major allele and show the positions of gRNA target sequences (blue underlined), PAM sequences (blue bold), gRNA-directed Cas9 cut sites (red arrows), and SNPs (red). The edited allele sequences show the expected locations and sizes of DNA fragment deletions. (C) PCR analysis demonstrating SNP microdeletions in bulk transfected LCLs. PCR products from non-transfected LCLs were analyzed for comparison (WT). Bulk populations with at least 1 band at the expected size were used for single cell cloning and are marked with an asterisk. Expected PCR product sizes (WT/Δ bp) rs62445866, 782/745; rs6964969, 765/716; rs6944602, 527/487; rs10264390, 772/731, 698/658. Panels are from images of either the same gel, or gels run separately that have been rearranged to reorder lanes.

We sought to determine whether any noncoding regions harboring SNPs in the rs58923657 LD block (r^2^ > 0.6) were functionally relevant to *IKZF1* expression. Since the rs58923657 LD block of SNPs resides within a B cell super-enhancer (S2B Fig), we focused on small (38–90 bp) regions that met at least 2 of the following criteria in GM12878 or other ENCODE LCLs: DNase I hypersensitivity, enhancer-associated chromatin state, and TF binding (Fig 1A and S1 Table) [36]. This strategy identified five candidate regions overlapping rs62445866, rs6964969, rs6944692, rs10264390, and rs17133807 (henceforth referred to by the SNP contained within each of the regions). Next, we examined whether these regions contribute to lymphoid *IKZF1* expression by generating isogenic LCLs that differed by homozygous deletions encompassing each individual SNP. We selected gRNAs with targets that minimized the size of each deletion, resulting in deletions of 38–90 bp (Fig 1B and S1A Fig). We confirmed the presence of SNP deletions in bulk transfected populations using PCR prior to single cell cloning (Fig 1C). Homozygous deletions in monoclonal cell lines were confirmed by Sanger sequencing (S1B Fig).

**Fig 2:**
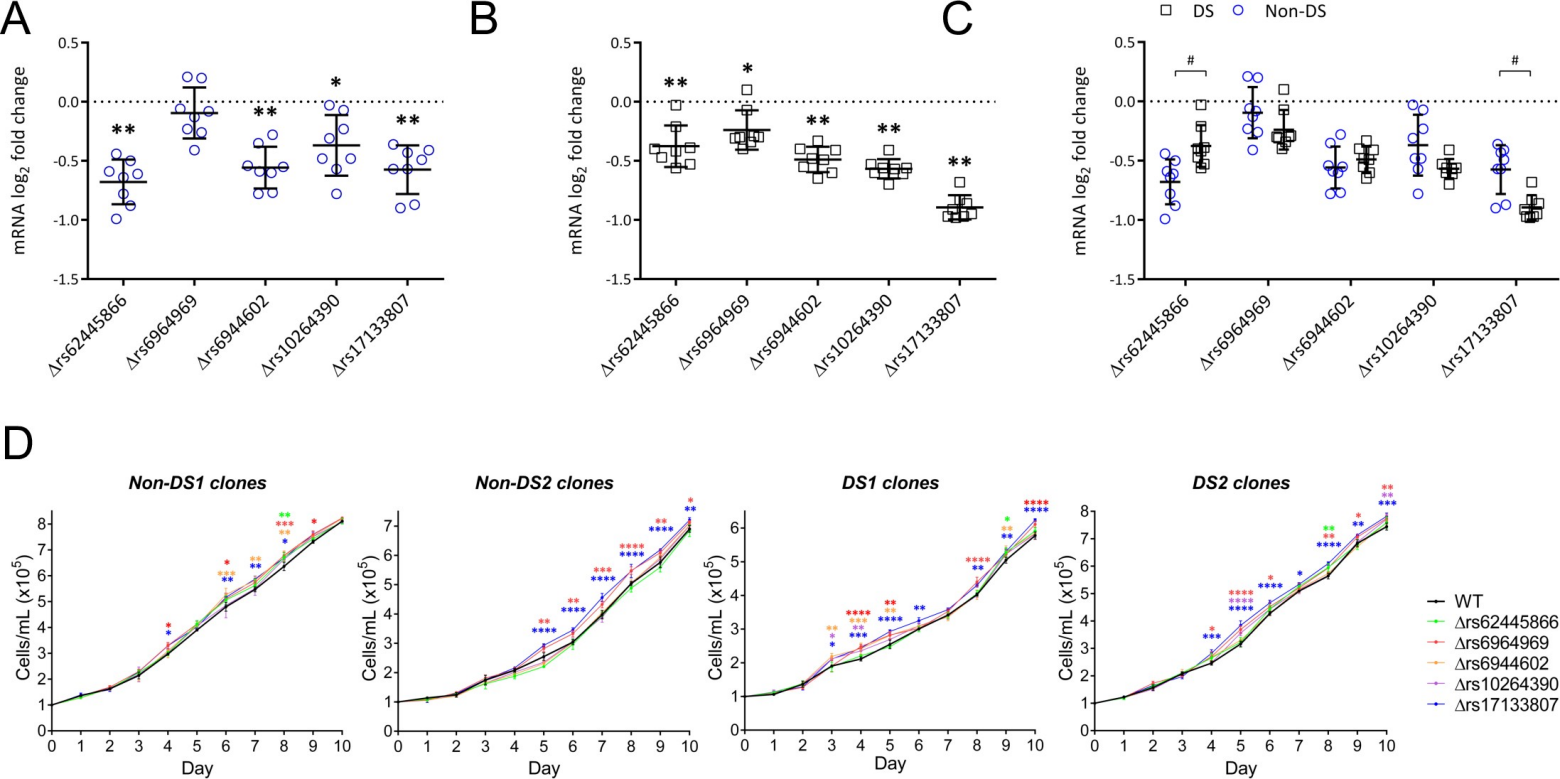
Homozygous microdeletions of SNPs in the rs58923657 non-risk haplotype result in significantly decreased *IKZF1* expression and increased proliferation in human LCLs. Expression of IKZF1 mRNA in (A) non-DS and (B) DS LCLs with CRISPR/Cas9 induced microdeletions of regions at rs62445866, rs6964969, rs6944602, rs10264390, and rs17133807. Dot plots show the log_2_ fold change of IKZF1 mRNA expression in SNP deletion clones normalized to WT clones in LCLs with non-risk allele status for each SNP. Results are mean ± SEM (n = 8 deletion clones per group). The differences between WT and SNP deletion groups were analyzed by one-way ANOVA. *P < 0.01; **P < 0.0001. (C) The effect on IKZF1 mRNA expression was compared for each SNP deletion between non-DS and DS clones. The differences were analyzed by one-way ANOVA. #*P <* 0.05. (D) Cellular proliferation in SNP deletion versus WT clones for 2 non-DS and 2 DS LCLs. Results are mean cell counts ± SEM (n = 3 clones per SNP deletion and 3 WT clones). Differences between WT and SNP deletion clones were analyzed by two-way ANOVA. *P < 0.05; **P < 0.01; ***P < 0.001; ****P < 0.0001.

In the non-DS LCLs, homozygous microdeletions encompassing 4 of 5 SNPs resulted in statistically significant reductions in IKZF1 mRNA levels compared with their WT counterparts (Δrs62445866 37.1%, Δrs6944602 31.7%, Δrs10264390 21.5%, and Δrs17133807 32.3% reduction; Fig 2A). In the DS LCLs, microdeletions encompassing each of the 5 SNPs resulted in statistically significant reductions in IKZF1 mRNA levels (Δrs62445866 22.4%, Δrs6964969 14.8%, Δrs6944602 28.6%, Δrs10264390 32.4%, and Δrs17133807 46.1% reduction; Fig 2B). In comparing the effect of each SNP deletion on *IKZF1* expression in DS and non-DS LCLs, Δrs62445866 resulted in a significantly greater reduction in non-DS LCLs, and Δrs17133807 resulted in a significantly greater reduction in DS LCLs (Fig 2C).

Since we previously showed that reducing IKZF1 expression by shRNA knockdown increased proliferation in LCLs [9], we next examined the effects of SNP microdeletions on proliferation in two DS and two non-DS LCLs. Δrs6964969 and Δrs17133807 resulted in the most consistent statistically significant increases in proliferation across all four DS and non-DS LCLs (Fig 2D). Δrs10264390 demonstrated some statistically significant increases in proliferation only in the DS LCLs. The other three SNP microdeletion clones demonstrated increases in proliferation at several but not all time points in both non-DS and DS LCLs.

### rs17133807 maps to a conserved enhancer

**Fig 3:**
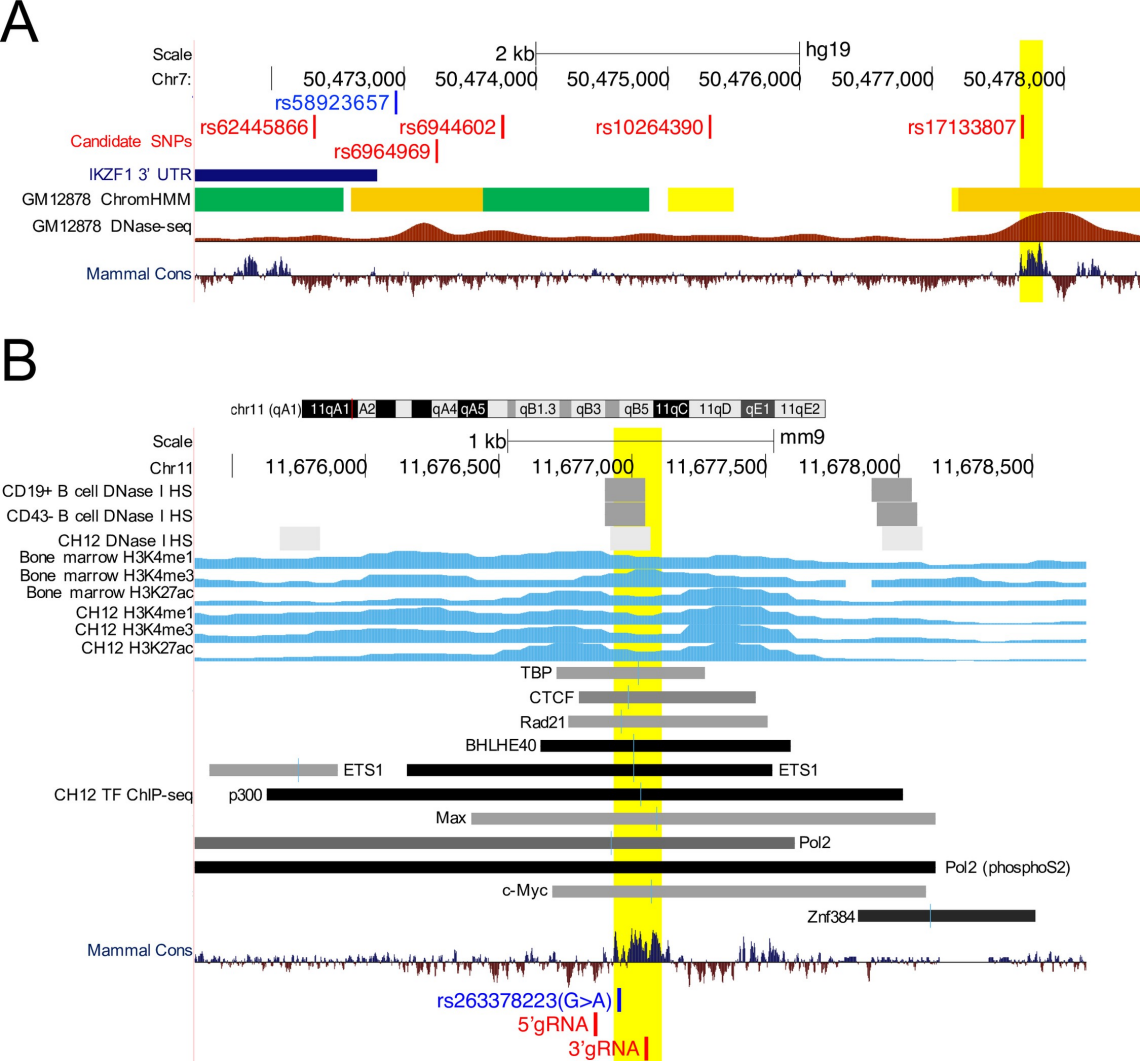
The *IKZF1* enhancer overlapping rs17133807 is conserved in mammalian species. (A) Epigenetic profile showing mammalian sequence conservation across the rs58923657 haplotype block. Tracks show *IKZF1* 3’ UTR position; GM12878 chromatin states; DNase-seq signal from GM12878; and mammalian sequence conservation with higher conservation scores above the axis reflecting increasing sequence conservation (see Fig 1A for additional details of track annotation). The conserved region encompassing rs17133807 is highlighted yellow. (B) Epigenetic profile of the orthologous mouse locus. Tracks show DNase I HS sites in mouse CD19^+^ and CD43^-^ B cells and the murine B lymphoblastoid cell line CH12; histone modification ChIP-seq data (H3K4Me1, H3K4Me3, H3K27ac) from mouse bone marrow and CH12; TF ChIP-seq data from CH12; mammalian sequence conservation; and the positions of mouse SNP rs263378223 (blue) and gRNA targets (red). The conserved region is highlighted yellow. All data are displayed in the UCSC genome browser (http://genome.ucsc.edu/).

Next, we evaluated sequence conservation of the rs58923657 haplotype block, with the goal of identifying highly conserved regions that could be further studied in the Dp16 mouse model of DS [26]. A region encompassing rs17133807 (hg19, chr7:50,477,670–50,477,845) is well-conserved across mammalian species (Fig 3A). DNase-seq, histone modification ChIP-seq, and TF factor ChIP-seq data from murine B cells, BM, and CH12 lymphoblastoid cells suggest the orthologous mouse region is also an *Ikzf1* enhancer in hematopoietic cells (Fig 3B). Furthermore, the mouse orthologue to rs17133807 is also a SNP (rs263378223; mm9, chr11:11,676,952) with the same major and minor alleles (G>A) as in humans (Fig 3B). To determine whether the region encompassing rs263378223 interacts with the *Ikzf1* promoter in murine lymphoid cells, we analyzed publicly available Hi-C data from CH12 cells (S2A Fig) [37]. The region encompassing mouse rs263378223 maps to the same topologically associated domain (TAD) as *Ikzf1*, suggesting that murine lymphoid cells have a 3D topology at *Ikzf1* that is conducive to enhancer-promoter interactions similar to what we previously described for human LCLs (S2B Fig) [9]. Based on this evidence indicating that the region encompassing mouse rs263378223 is a lymphoid *Ikzf1* transcriptional enhancer, we proceeded with further functional studies. We were unable to identify conserved regions corresponding to the other 4 candidate SNPs in the *IKZF1* haplotype block that we had evaluated in human LCLs.

### Functional studies of the conserved human rs17133807 enhancer region in mice

**Fig 4:**
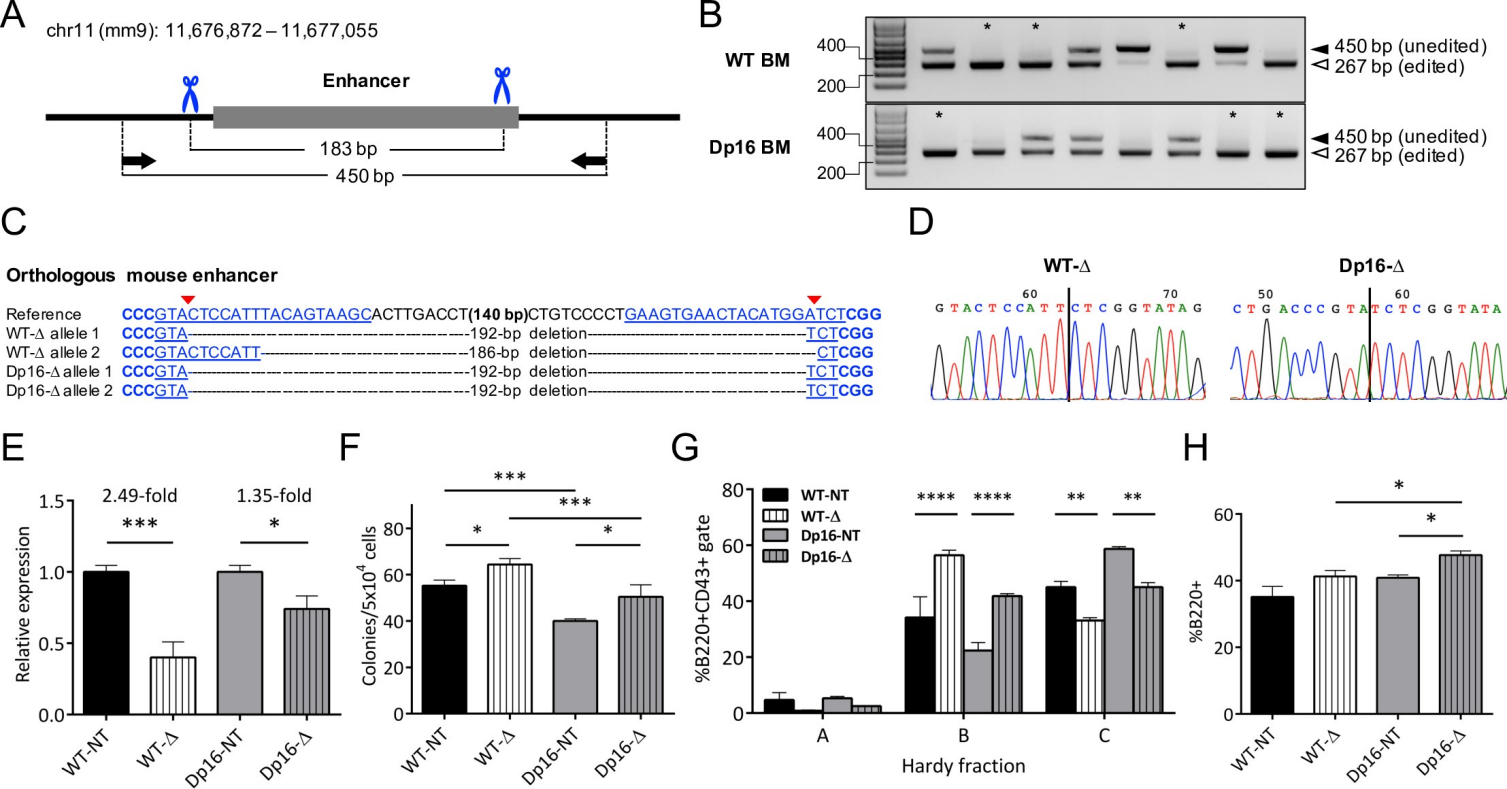
Homozygous deletion of the orthologous murine *Ikzf1* enhancer results in significantly decreased *Ikzf1* expression, increased cell proliferation, and altered B cell differentiation. (A) Schematic of the orthologous enhancer region on mouse chr11 showing gRNA-directed Cas9 cut sites (blue scissors) and primer binding sites (black arrows). (B) PCR analysis of CRISPR/Cas9mediated deletions at the orthologous enhancer in WT and Dp16 BM HSCs. Samples with at least 1 band at the expected size and no unedited band (*) were sequenced. (C) Alignment of representative Sanger sequencing results from edited Dp16 and WT samples. The positions of gRNA target sequences (blue underlined), PAM sequences (blue bold), and gRNA-directed Cas9 cut sites (red arrows) are shown. (D) Representative chromatograms from samples in (C) are shown with the repaired DNA junctions marked (vertical line). (E) Relative Ikzf1 mRNA expression in c-Kit^+^Sca-1^+^CD34^-^ HSCs with homozygous enhancer deletion (Δ) was analyzed by qRT-PCR. Bars show mean ± SEM normalized to non-targeting (NT) gRNA-expressing controls. Results were analyzed by Student’s two-tailed t-test. (F) Pre-B lymphoid colony forming assays were performed using transduced BM HSCs. ZsGreen^+^RFP^+^ colonies for NT and Δ samples were counted after incubating for 7 days in pre-B lymphoid-promoting methylcellulose. Bars show mean colony counts ± SEM. (G) B cell differentiation was analyzed in Δ and NT HSCs by Hardy fraction analysis. Bars show mean ± SEM percentages of cells in each Hardy fraction. (H) The percentage of B220^+^ lymphocytes in Δ and NT HSC co-cultures was measured by flow cytometry. Bars show mean ± SEM percentages of B220^+^ cells. Results from (E)–(H) are from independently transduced HSC harvests from n = 3 mice. Results in (E) and (H) were analyzed by Student’s t-test. Results in (F) and (G) were analyzed by two-way ANOVA with Tukey's multiple comparisons test. * *P* < 0.05; ** *P* < 0.01; *** *P* < 0.001; **** *P* < 0.0001.

Parallel to our generation of genome-edited SNP microdeletions in human LCLs, we utilized CRISPR/Cas9-induced microdeletions to study effects of the putative enhancer encompassing rs263378223 on *Ikzf1* expression in mouse HSCs. We selected dual gRNAs to create a 183 bp deletion encompassing the B cell DNase I hypersensitive region harboring rs263378223 (Fig 3B) and cloned them into the same vectors used for the LCL experiments (Fig 4A and S2 Table). We used these plasmids to generate lentivirus to improve transgene delivery and cell viability, and to allow prolonged tracking of enhancer microdeletion mutants stably expressing both fluorescent markers. We achieved exceptional genome editing frequencies in transduced HSCs using this strategy, with nearly all samples showing evidence of a microdeletion by PCR amplification (Fig 4B). Sanger sequencing of TA-cloned PCR products was performed to confirm enhancer deletion in transduced BM HSC samples (representative examples are shown in Fig 4C and 4D).

Homozygous deletion of the region encompassing rs263378223 (Δrs263378223) resulted in significant reductions of Ikzf1 mRNA levels in transduced HSCs, with 1.35-fold reduction in Dp16 and 2.49-fold reduction in WT littermate HSCs (Fig 4E), confirming that the locus acts as a distal *Ikzf1* transcriptional enhancer in mouse HSCs. Next, we assessed whether HSCs with Δrs263378223 demonstrated alterations in B-lineage proliferation and differentiation. To assess proliferation, we performed colony forming assays with transduced HSCs in lymphoid-promoting medium. After 7 days, HSCs with Δrs263378223 produced significantly more lymphoid colonies in both the Dp16 (*P <* 0.05) and WT (*P <* 0.05) genotypes (Fig 4F). At baseline, Dp16 HSCs produced significantly fewer colonies than WT (*P <* 0.001, Fig 4F), which is in accordance with known hematologic deficiencies in DS such as defects in lymphocyte composition and function [38], but there was no significant difference in the relative fold increase in colony counts between Dp16 and WT HSCs with Δrs263378223 (1.26 versus 1.17 fold increase, *P* = 0.30). We examined B-lineage differentiation by performing Hardy fraction analysis [35] of transduced HSCs co-cultured with OP9 cells in lymphoid-promoting medium. Both Dp16 and WT Δrs263378223 HSCs demonstrated a shift toward more immature B-lineage progenitors, with significantly increased Hardy fraction B (pro-B) cells (*P* < 0.0001) and decreased Hardy fraction C (late pro-B) cells (*P* < 0.01) (Fig 4G). The changes in Hardy fractions did not differ significantly between the Dp16 and WT genotypes. In addition to Hardy fraction analysis, we also evaluated the percentage of B220^+^ cells (Fig 4H). Δrs263378223 resulted in a significant increase in B220^+^ cell production in Dp16 HSCs (*P =* 0.011) but not in WT HSCs (*P =* 0.164). Collectively, these results suggest that the region harboring rs263378223 is a B-lineage *Ikzf1* enhancer that exerts functional effects on Ikzf1 transcription and B cell proliferation and differentiation.

## Discussion

*IKZF1* is a master regulator of lymphoid development and is consistently one of the top association signals in GWAS of ALL susceptibility [1, 2, 9]. However, studies are limited that define the molecular mechanisms underlying GWAS-identified risk loci, including *IKZF1*. Several prior studies have demonstrated an association between *IKZF1* risk loci and IKZF1 expression [1, 39–41]. However, these studies have not interrogated the specific SNPs and overlapping regulatory regions that functionally mediate decreased expression, nor have they confirmed other functional consequences in lymphoid cells. We previously performed initial functional characterization of the *IKZF1* susceptibility locus [9]. We used ENCODE epigenetic datasets to identify five candidate regulatory SNPs in LD with rs58923657 (r^2^ > 0.6) with a plausible basis for mediating ALL risk through effects on TF binding, chromatin accessibility, and/or epigenetic modification. We then demonstrated that the haplotype block encompassing these SNPs exhibits allele-specific regulatory activity and that the risk allele haplotype block exhibits reduced enhancer activity and differential protein binding.

In this study, we used CRISPR/Cas9-mediated microdeletions in hematopoietic cells to further interrogate the impact of the regulatory regions overlapping these five candidate functional SNPs on IKZF1 mRNA expression and B cell proliferation. Additionally, we examined the effects of a highly conserved murine enhancer encompassing rs263378223 on Ikzf1 expression and B cell proliferation and differentiation. We found that each region harboring one of the *IKZF1* SNPs demonstrated some degree of *IKZF1* enhancer activity, and that there was some variability based on DS versus non-DS genetic background. All five SNP microdeletions significantly decreased IKZF1 expression in DS LCLs, and 4 of 5 decreased expression in non-DS LCLs. The reduction of IKZF1 expression was similar in DS and non-DS LCLs for 3 of the SNP microdeletions, greater in DS LCLs for Δrs17133807, and greater in non-DS for Δrs62445866. These SNPs reside in a region that is over 150 kb from the *IKZF1* promoter, suggesting the mechanism of this risk locus involves long-range enhancer activity.

We utilized the Dp16 mouse model of DS to conduct further studies of an enhancer overlapping rs17133807, which is highly conserved in mice and overlaps an orthologous mouse SNP, rs263378223. This approach has been useful in functional studies of other GWAS loci [42, 43], and may provide a useful tool to evaluate the effects of the *Ikzf1* risk locus in disease-relevant tissues over the lifetime of a model organism. Δrs263378223 resulted in significantly decreased Ikzf1 expression for both WT and Dp16 HSCs, confirming the region acts as an active *Ikzf1* transcriptional enhancer. Δrs263378223 resulted in a greater reduction in Ikzf1 expression in WT HSCs compared to Dp16 HSCs, whereas the effect on expression was greater in DS compared to WT LCLs for Δrs17133807. This may be related to differences in the size of the regions deleted in the LCLs and HSCs (40 vs 183 bp, respectively), or to uncharacterized, intrinsic differences in the mediation of enhancer activity between primary murine HSCs and transformed human LCLs. Nonetheless, despite differences in the magnitude of the reduction in Ikzf1 expression in WT versus Dp16 HSCs, Δrs263378223 had similar impact on WT and Dp16 B-progenitors in functional assays. Specifically, Δrs263378223 resulted in significantly increased B-lineage colony formation, and a relative increase in pro-B and decrease in late pro-B phase progenitors in both WT and Dp16 cells, and a significant increase in B220^+^ cells in Dp16 but not WT HSCs (Fig 4).

One limitation of this study is that we did not directly compare the effects of the risk versus non-risk alleles of each SNP by using CRISPR/Cas9 single base editing. Generation of isogenic allelic variant LCL clones is another potential strategy [44], but was not feasible in our LCLs due to very low efficiency of the required homology-dependent repair. However, generating biallelic microdeletions encompassing potentially functional SNPs is a viable alternative strategy to efficiently assess whether regions associated with a susceptibility locus play a role in gene expression or cellular processes associated with risk [45]. The rs58923657-tagged risk locus is bound by numerous TFs in GM12878, with the majority binding at rs17133807 being relevant to lymphocyte development, transcription, and enhancer maintenance. Future studies should investigate whether the risk allele of rs17133807 influences binding of these factors, and whether binding differs in the DS genetic background.

In sum, our findings provide a detailed interrogation of the functional impact of variation at the *IKZF1* risk locus in the DS and non-DS genetic backgrounds, demonstrating functional effects of microdeletions of risk allele regions on gene expression, B-lineage proliferation, and differentiation, in both humans and mice. In particular, the enhancer encompassing rs17133807 was associated with the greatest reduction of IKZF1 expression in DS LCLs, the most consistent increase in proliferation in serial cell counts, and similar effects for the orthologous murine enhancer as well as effects in this system on B-lineage differentiation. The fact that this locus demonstrated the most potent and varied effects is unsurprising, since it overlies a binding site for numerous transcription factors (Fig 1A). Overall, these findings confirm the functional importance of the *IKZF1* locus in both DS and non-DS models. There is some variability according to DS status, but most effects were similar overall in both genetic backgrounds, suggesting that other factors may play a greater role in explaining the increased risk of ALL associated with DS.

## Supporting information

S1 Fig CRISPR/Cas9 mediated microdeletions of SNPs in the rs58923657 non-risk haplotype block in human LCLs. (A) Representative alignments of Sanger sequencing results from non-DS and DS LCL SNP microdeletion clones. The positions of gRNA target sequences (blue and underlined), PAM sequences (blue and bold), and sizes of fragment deletions are shown for each allele. (B) Representative chromatograms from samples in (A) are shown with the repaired DNA junctions marked (vertical line).
(TIF)

S2 Fig 3D topology of *IKZF1* in human and mouse lymphoblastoid cell lines. Chromatin spatial organization of (A) mouse chr11 11.0–12.0 MB and (B) human chr7 from 50.0–50.6 MB. Tracks for (A) show a Hi-C heatmap of chromatin contact frequencies in CH12; CH12 CTCF ChIP-seq; Refseq genes; TAD determined by the Arrowhead algorithm (PMID: 25497547); pro-B cell super-enhancers (21); and the enhancer encompassing rs263378223. Tracks for (B) show a Hi-C heatmap of chromatin contact frequencies in GM12878; GM12878 CTCF ChIP-seq; Refseq genes; TAD determined by the Arrowhead algorithm (37); CD19+ B-cell super-enhancers (21); and the rs58923657 haplotype block. Black dashed lines and circle indicate chromatin looping domains.
(TIF)

S1 Table Oligonucleotide sequences for gRNA cloning. (XLSX)

S2 Table Primer sequences. (XLSX)

S3 Table Candidate SNPs for functional analysis. (XLSX)

S1 Raw images (PDF)
