# Supplementary figures and images for: Enhancer polymorphisms at the IKZF1 susceptibility locus for acute lymphoblastic leukemia impact B-cell proliferation and differentiation in both Down syndrome and non-Down syndrome genetic backgrounds
Source: PLoS One. 2021 Jan 7;16(1):e0244863. doi: 10.1371/journal.pone.0244863 (PMC7790404; doi:10.1371/journal.pone.0244863)

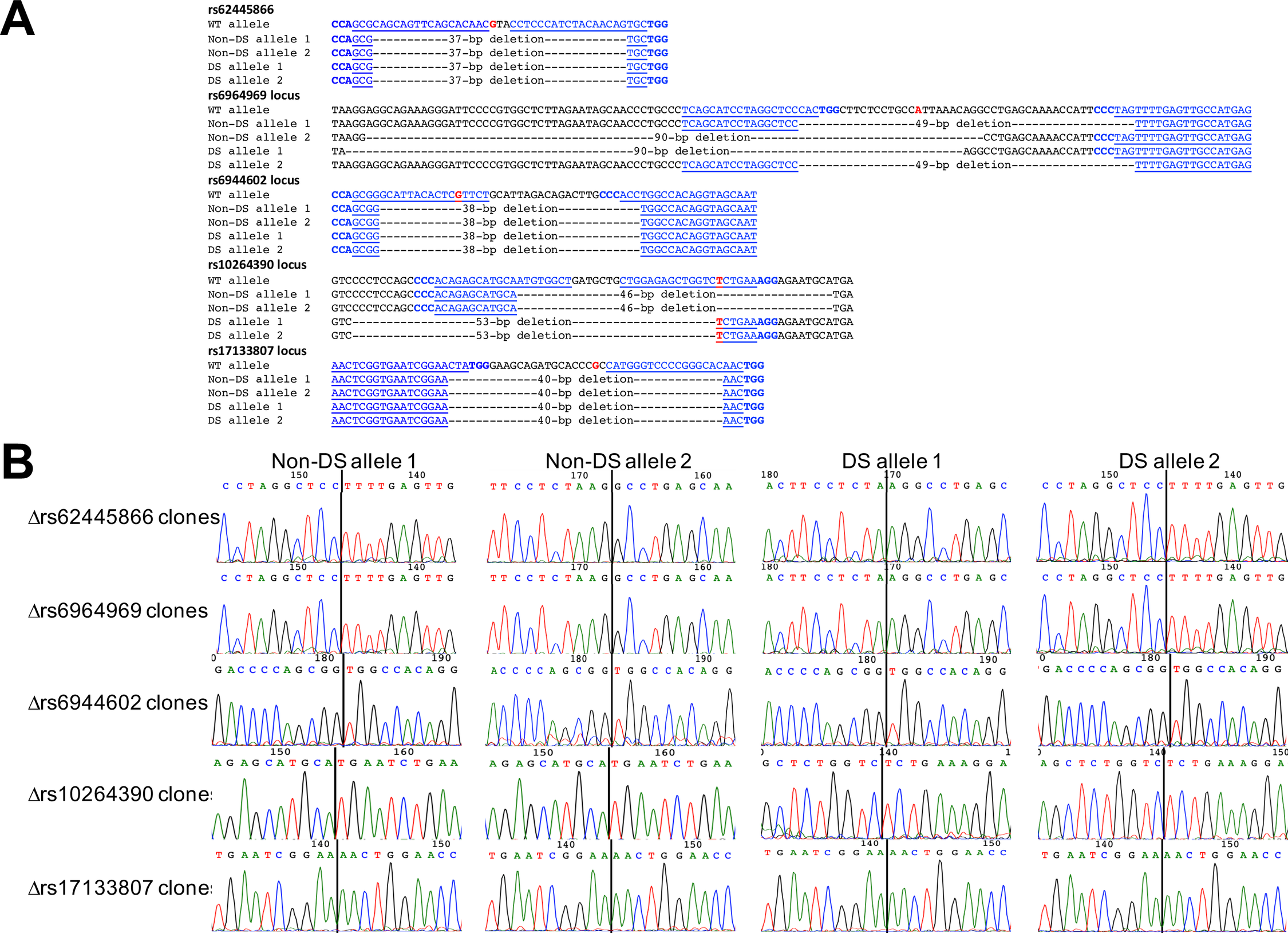

Supplement: S1 Fig — (A) Representative alignments of Sanger sequencing results from non-DS and DS LCL SNP microdeletion clones. The positions of gRNA target sequences (blue and underlined), PAM sequences (blue and bold), and sizes of fragment deletions are shown for each allele. (B) Representative chromatograms from samples in (A) are shown with the repaired DNA junctions marked (vertical line). (TIF) [file pone.0244863.s001.tif]

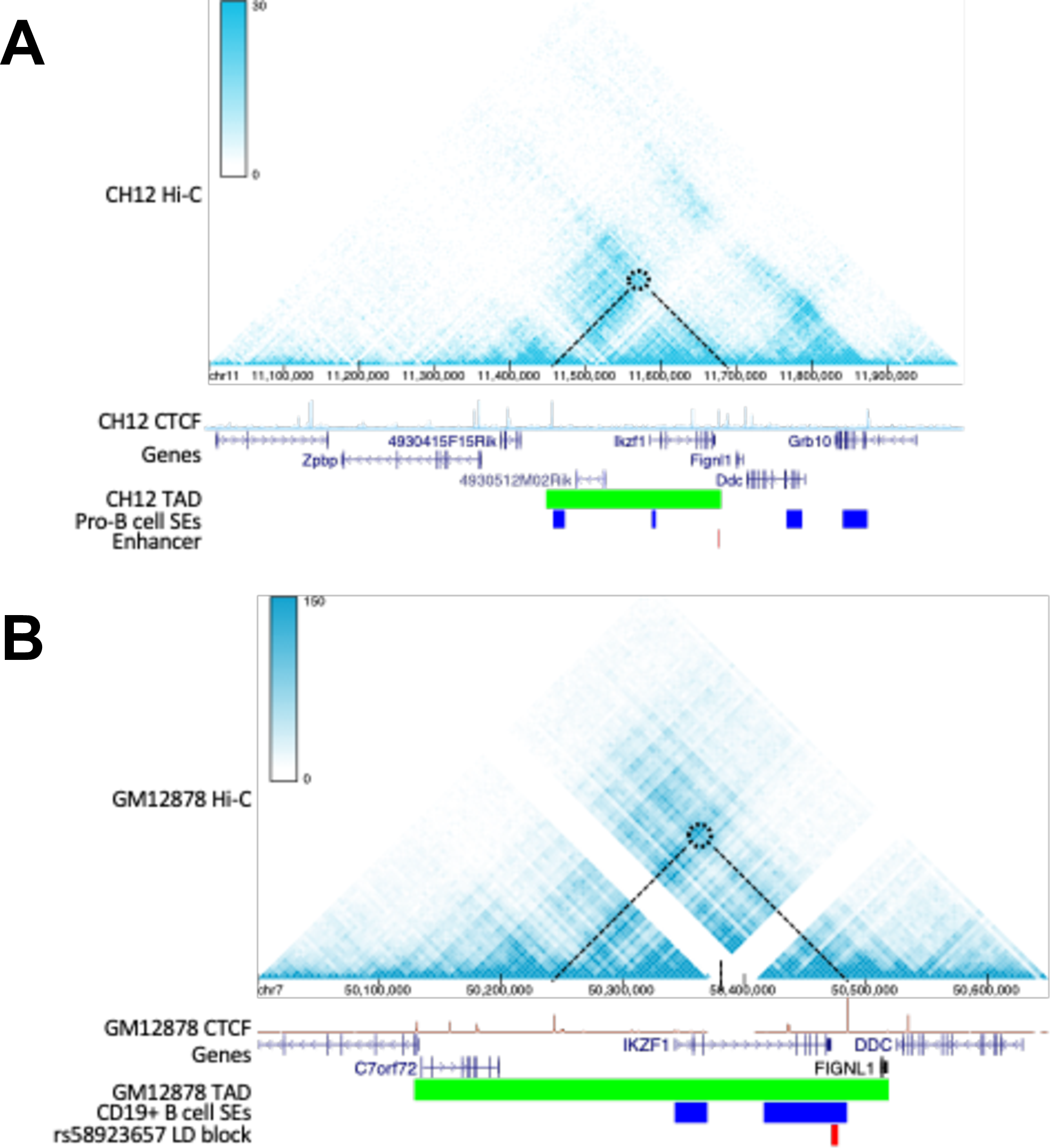

Supplement: S2 Fig — Chromatin spatial organization of (A) mouse chr11 11.0–12.0 MB and (B) human chr7 from 50.0–50.6 MB. Tracks for (A) show a Hi-C heatmap of chromatin contact frequencies in CH12; CH12 CTCF ChIP-seq; Refseq genes; TAD determined by the Arrowhead algorithm (PMID: 25497547); pro-B cell super-enhancers (21); and the enhancer encompassing rs263378223. Tracks for (B) show a Hi-C heatmap of chromatin contact frequencies in GM12878; GM12878 CTCF ChIP-seq; Refseq genes; TAD determined by the Arrowhead algorithm (37); CD19+ B-cell super-enhancers (21); and the rs58923657 haplotype block. Black dashed lines and circle indicate chromatin looping domains. (TIF) [file pone.0244863.s002.tif]
