# Supplementary material for: Enhancer polymorphisms at the IKZF1 susceptibility locus for acute lymphoblastic leukemia impact B-cell proliferation and differentiation in both Down syndrome and non-Down syndrome genetic backgrounds
Source: PLoS One. 2021 Jan 7;16(1):e0244863. doi: 10.1371/journal.pone.0244863 (PMC7790404; doi:10.1371/journal.pone.0244863)

Non-DS LCL bulk transfected populations, figure 1C, imaged with Bio-Rad Versadock

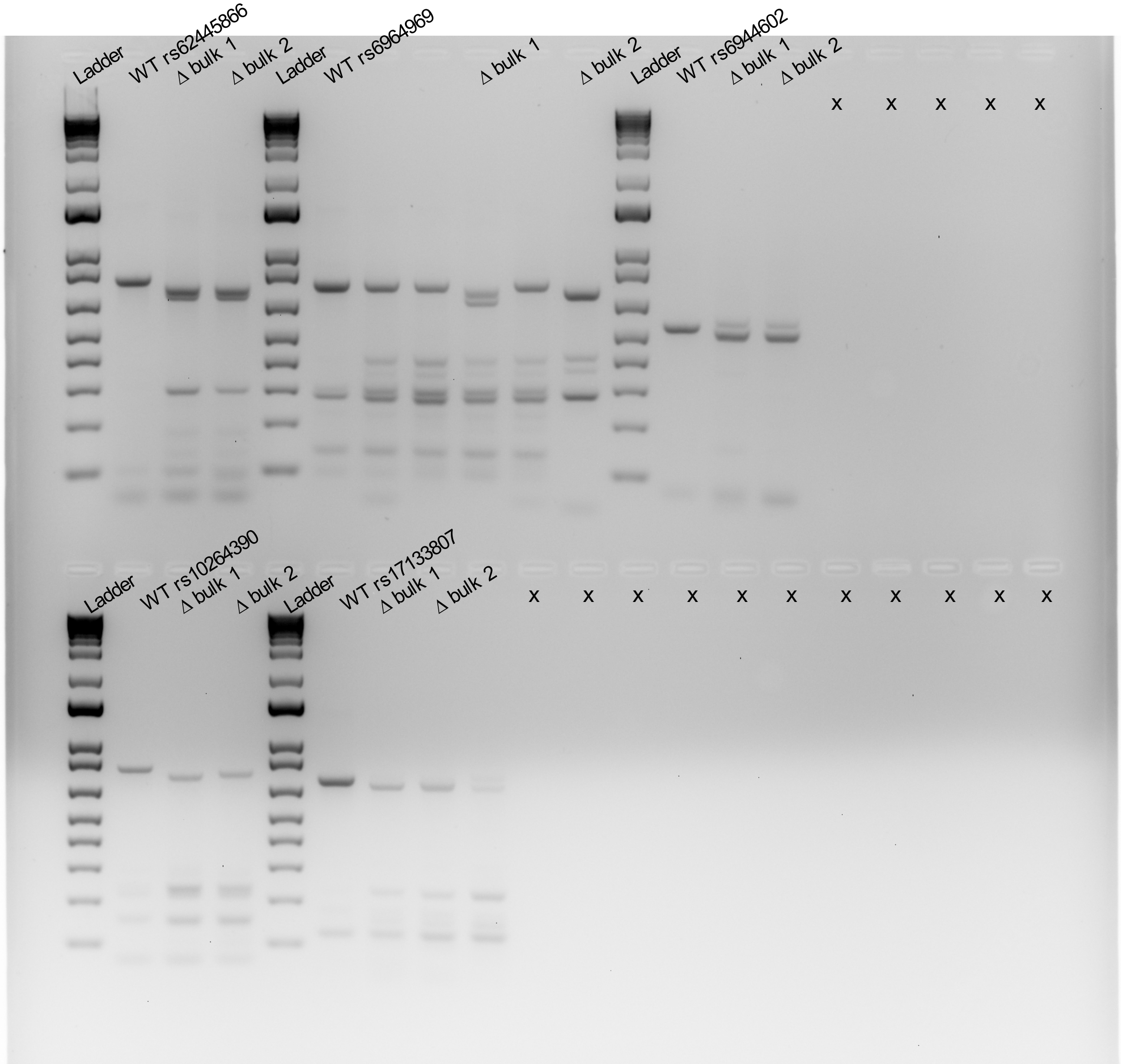

DS LCL bulk transfected populations, figure 1C, imaged with Bio-Rad Versadock

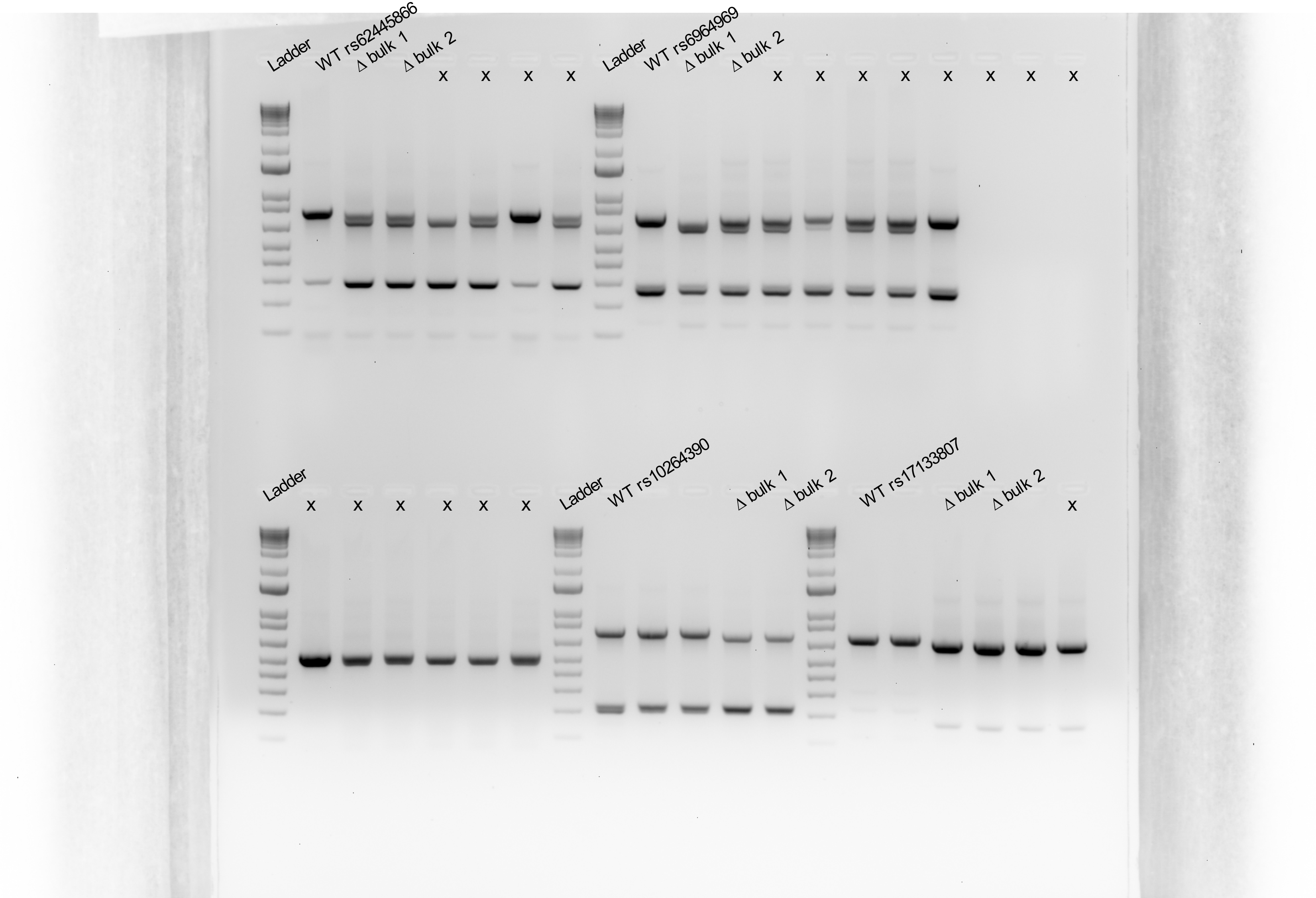

DS LCL bulk transfected populations, figure 1C, imaged with Bio-Rad Versadock

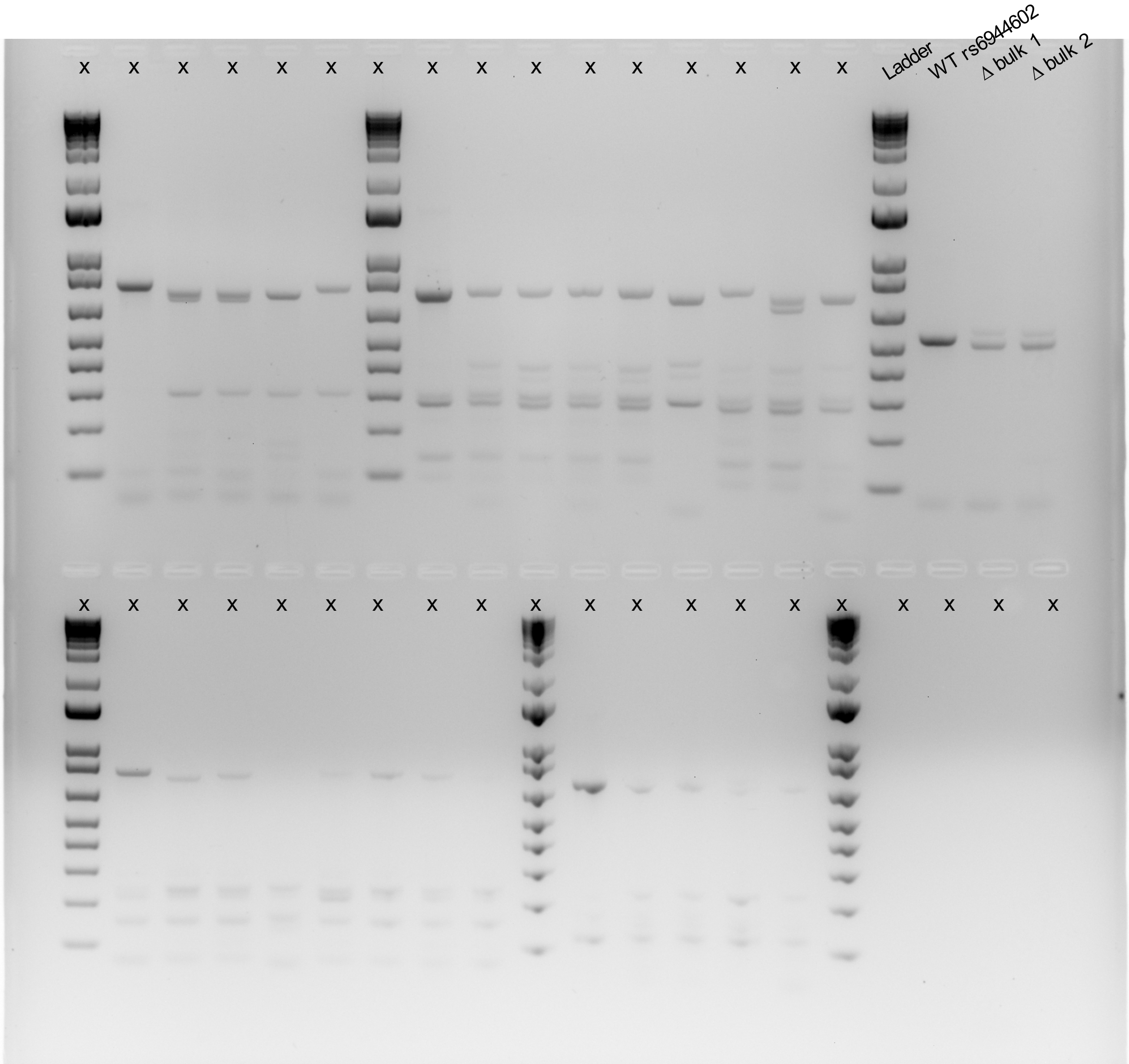

Transduced mouse BM HSCs, figure 4B, imaged with Bio-Rad Versadock

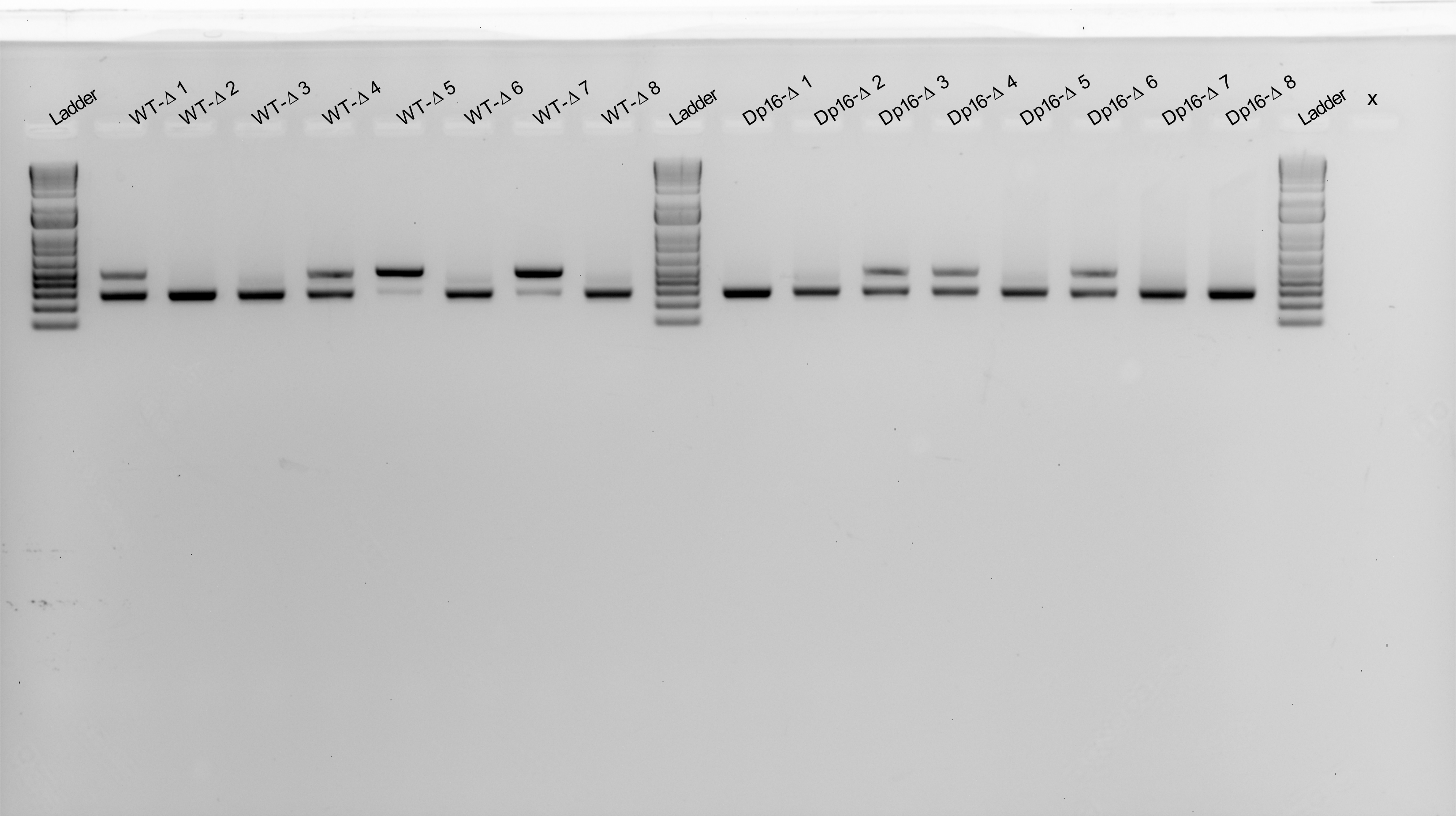

Supplement: S1 Raw images — (PDF) [file pone.0244863.s006.pdf]
